# Cross-Cohort, Renal Compartment-Aware Transcriptomic Assessment of Lycii Fructus-Annotated Targets in Diabetic Kidney Disease

**DOI:** 10.3390/cimb48080829

**Published:** 2026-08-14

**Authors:** Jinhao Zou, Xiaowei Tian, Ye Sun, Siyi Wang

**Affiliations:** 1 Key Laboratory of Dryness Syndrome in Chinese Medicine, Ministry of Education, Ningxia Medical University, Yinchuan 750004, China; 2 College of Traditional Chinese Medicine, Ningxia Medical University, Yinchuan 750004, China; 3 College of Pharmacy, Ningxia Medical University, Yinchuan 750004, China

**Keywords:** Lycii Fructus, *Lycium barbarum* L., diabetic kidney disease, renal transcriptomics, target-set enrichment, molecular docking

## Abstract

Diabetic kidney disease (DKD) differs transcriptionally across renal compartments. We assessed whether Lycii Fructus-annotated targets showed reproducible DKD-associated changes and enrichment beyond matched expectations. Linked glomerular (GSE30528) and tubulointerstitial (GSE30529) cohorts were used for discovery; GSE96804 and platform-specific GSE104954 subsets provided external assessment. Phenotype-independent probe aggregation preceded limma analysis. Of 106 standardized HERB targets, 91 were measurable. Specificity was tested by hypergeometric analysis and 10,000 expression- and publication-matched permutations; external evidence was graded symmetrically. Thirty-six targets met the primary criterion in at least one compartment, but the target set was not enriched. CASP8 and VEGFA met Tier A external criteria, whereas ADRB2 and SLC6A2 met Tier B criteria. At the stricter |log_2_FC| ≥ 0.585 threshold, ADRB2 passed in both discovery compartments, VEGFA only in glomeruli, and CASP8/SLC6A2 in neither. STRING analysis used the measured-gene background. Reference redocking reproduced carazolol in ADRB2 and atomoxetine in SLC6A2 (RMSD, 1.08 and 1.09 Å). Thus, the four genes are reproducible DKD-perturbed candidates carrying Lycii Fructus annotations, not evidence of a Lycii Fructus-specific mechanism; they warrant targeted pharmacological testing.

## 1. Introduction

Diabetic kidney disease (DKD) is a major cause of chronic kidney disease and kidney failure. Its progression involves glomerular endothelial injury, podocyte dysfunction, extracellular-matrix remodeling, tubular stress, inflammation, and metabolic disturbance. Because glomerular and tubulointerstitial lesions are not transcriptionally interchangeable, analyses that merge renal compartments may obscure biologically relevant differences [1,2,3,4,5,6,7,8]. Public renal transcriptomic cohorts therefore provide an opportunity to evaluate candidate targets at the tissue-compartment level and across independently collected cohorts.

Lycii Fructus (Gouqizi), the dried mature fruit of *Lycium barbarum* L. recorded in the 2025 edition of the Chinese Pharmacopoeia, is used as both a medicinal and edible material in China. Experimental studies of *Lycium barbarum* polysaccharides and related extracts have reported effects on oxidative stress, inflammatory signaling, glucose and lipid metabolism, and renal injury in diabetic models [9,10,11,12,13,14,15]. These findings concern defined extracts or polysaccharide fractions and should not be treated as direct evidence for the clinical effect of the whole crude drug. Meanwhile, databases such as HERB connect herbal medicines to large numbers of genes through heterogeneous evidence, including experiments, text mining, and curated associations [16,17,18]. A gene’s presence in such a database does not by itself demonstrate herb specificity or disease relevance.

Many network-pharmacology studies begin by intersecting a broad herb-target list with disease-associated genes and then interpret the overlap as mechanistic support. This procedure can preferentially recover well-studied and highly expressed genes. It rarely tests whether the observed overlap exceeds that expected for targets with comparable expression and publication characteristics. The resulting biological narrative may therefore be traceable yet non-specific.

The present study addressed this problem through a cross-cohort, renal compartment-aware design. We first identified DKD-associated transcriptional changes among HERB-annotated Lycii Fructus targets in linked glomerular and tubulointerstitial discovery datasets. We then tested target-set specificity using analytical and matched-permutation null models, assessed candidate reproducibility in external cohorts under symmetric evidence rules, examined expression quality and probe behavior, used measured-background STRING analysis, and calibrated molecular docking for mechanistic contextualization. The aim was target prioritization, not confirmation of efficacy or causality.

## 2. Materials and Methods

### 2.1. Study Design and Transcriptomic Cohorts

Human renal transcriptomic data were obtained from the Gene Expression Omnibus (GEO) [19,20]. GSE30528 comprised 22 glomerular expression profiles (9 DKD and 13 control), whereas GSE30529 comprised 22 tubulointerstitial expression profiles (10 DKD and 12 control). Thus, the discovery datasets contained 44 expression profiles. Participant-identifier auditing showed that these two discovery datasets were linked: nine individuals (five DKD and four controls) contributed to both compartments, so the 44 profiles represented 35 unique discovery participants rather than 44 independent participants. The datasets were therefore treated as compartment-specific discovery views rather than independent replications (Supplementary Table S1; Supplementary Data Workbook, sheets “DiscoveryIndividuals” and “ParticipantOverlap”).

External assessment used GSE96804 and GSE104954. GSE96804 included glomerular samples from 41 participants with DKD and 20 histologically unaffected renal-cortex controls obtained from tumor nephrectomy specimens [21]. GSE104954 included tubulointerstitial profiles on two platforms. The GPL22945 subset contained 7 DKD and 18 living-donor control samples and served as the principal tubulointerstitial external cohort. The GPL24120 subset contained 10 DKD and 3 living-donor control samples and was retained as a sensitivity cohort because of its small control group and platform difference [22,23,24]. No new human participants or biological specimens were recruited.

**Figure 1:**
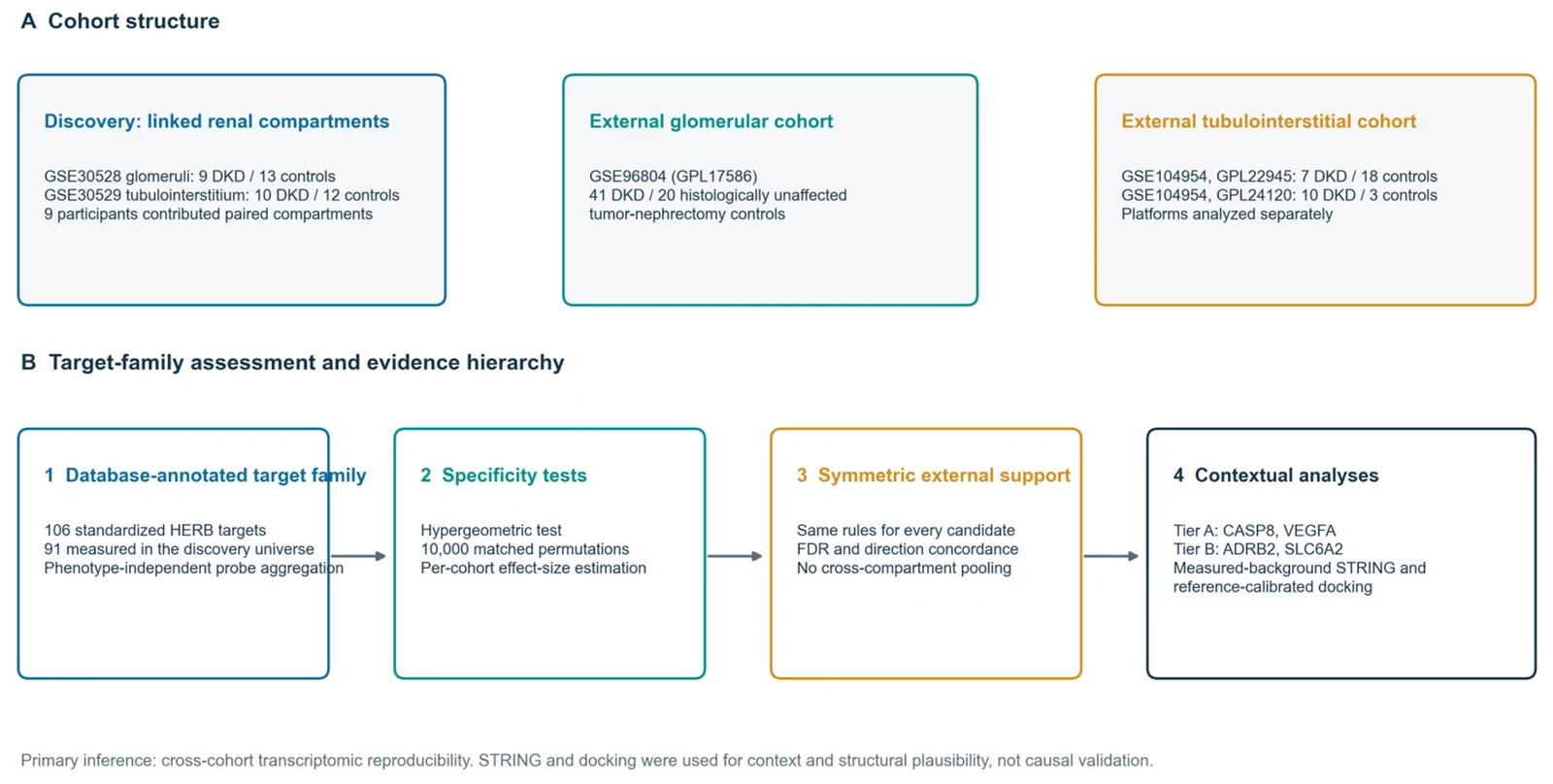
Study design and evidence hierarchy. The discovery cohorts represent linked glomerular and tubulointerstitial samples and were not treated as independent biological cohorts. External cohorts were analyzed by compartment and platform. The primary inference concerned transcriptomic reproducibility; STRING and docking supplied contextual and structural evidence.

Publicly recoverable clinical descriptors were available only at the aggregate cohort level. In GSE30528, controls versus DKD cases included 5/13 versus 5/9 female participants, had mean ages of 51.38 ± 12.01 versus 64.00 ± 13.56 years, mean estimated glomerular filtration rates (eGFRs) of 80.91 ± 23.42 versus 31.08 ± 13.36 mL/min, and mean dipstick-proteinuria scores of 0.69 ± 0.85 versus 2.55 ± 1.74. Corresponding values for GSE30529 were 5/12 versus 6/10 female participants, ages of 54.08 ± 13.81 versus 63.50 ± 15.64 years, eGFRs of 73.77 ± 21.08 versus 21.85 ± 11.54 mL/min, and dipstick-proteinuria scores of 0.40 ± 0.84 versus 3.40 ± 0.84. In GSE96804, controls versus DKD cases included 14/6 versus 29/12 male/female participants, had mean ages of 43.2 ± 6.52 versus 46.9 ± 7.70 years, mean eGFRs of 100.23 ± 10.5 versus 63.16 ± 22.12 mL/min/1.73 m^2^, and proteinuria shown as “—” for controls versus 2.53 ± 1.20 g/24 h for DKD [21,22,23,24]. Diabetes duration was not publicly reported for these exact cohorts. For the exact GSE104954 platform-specific DKD/control subsets analyzed here, age, sex, eGFR, proteinuria, and disease-duration values could not be linked from the public GEO records or associated article. Because individual-level clinical data were not consistently available across cohorts, these descriptors were used only to characterize the samples and were not included as covariates. The characteristics and analysis roles of the five dataset/platform subsets are summarized in Table 1. The overall study design and evidence hierarchy are shown in Figure 1.

**Table 1:** Transcriptomic cohorts and analysis roles.

Dataset	Compartment	Platform	DKD, n	Control, n	Control Source	Role
GSE30528	Glomerular	GPL571	9	13	Non-diabetic kidney tissue	Discovery
GSE30529	Tubulointerstitial	GPL571	10	12	Non-diabetic kidney tissue	Discovery
GSE96804	Glomerular	GPL17586	41	20	Histologically unaffected tumor-nephrectomy tissue	External assessment
GSE104954	Tubulointerstitial	GPL22945	7	18	Living-donor kidney tissue	External assessment
GSE104954	Tubulointerstitial	GPL24120	10	3	Living-donor kidney tissue	Sensitivity assessment

DKD, diabetic kidney disease. GSE30528 and GSE30529 are linked renal-compartment datasets; nine participants (four controls and five DKD cases) contributed samples to both datasets. The two discovery datasets therefore comprise 44 expression profiles from 35 unique participants. Across all five dataset/platform subsets, 143 denotes expression profiles (44 discovery plus 99 external), not independent participants. The two GSE104954 platforms were analyzed separately. Publicly recoverable aggregate clinical descriptors are reported in Section 2.1; exact participant-level age, sex, eGFR, proteinuria, and diabetes-duration data could not be linked to the two GSE104954 platform subsets.

### 2.2. Preprocessing and Differential Expression

Series matrices and platform annotations were downloaded from GEO. Probe annotations were standardized to current human gene symbols. When multiple probes represented one gene, probe-level expression was aggregated without reference to phenotype labels, thereby preventing outcome-informed probe selection. Differential expression was estimated separately within each cohort using limma with empirical-Bayes variance moderation [25]. The primary discovery criterion was false-discovery rate (FDR) < 0.05 and |log_2_ fold change (log_2_FC)| ≥ 0.20. A stricter |log_2_FC| ≥ 0.585 criterion was evaluated separately as a sensitivity analysis and did not replace the primary criterion. The lower primary effect-size threshold was used to accommodate the compressed dynamic range of microarray measurements; inference did not rely on this threshold alone because external consistency, effect estimates, and null-model testing were evaluated separately (Supplementary Figure S3; Supplementary Table S5; Supplementary Data Workbook, sheet “StrictThreshold”).

### 2.3. Lycii Fructus Target Set and Measured Universe

Lycii Fructus-associated targets were obtained from HERB using the Fructus Lycii entry and the *L. barbarum*-specific entry. Symbols were standardized, duplicates were removed, and mapping decisions were recorded. The union comprised 106 standardized human targets. The discovery measured universe consisted of 12,501 genes available after harmonization in both discovery compartments; 91 of the 106 targets were measurable in this universe. A target was considered discovery positive when it met the primary differential-expression criterion in at least one compartment. Candidates significant in both compartments were additionally classified by direction concordance (Supplementary Data Workbook, sheets “HerbTargets106” and “DiscoveryCandidates36”).

### 2.4. Target-Set Specificity Tests

Two complementary null models were used. First, one-sided hypergeometric tests compared the observed number of Lycii Fructus targets among four discovery outcomes: glomerular differential expression, tubulointerstitial differential expression, differential expression in either compartment, and same-direction differential expression in both compartments. The 12,501-gene measured universe was used as the denominator.

Second, each measurable target was matched to non-target genes on average expression and the number of unique PubMed identifiers linked through MyGene GeneRIF records. This GeneRIF-derived quantity was used as a publication-attention proxy and was not interpreted as a total publication count. For each outcome, 10,000 matched gene sets of equal size were sampled with seed 20260729. The empirical one-sided *p* value was calculated as (1 + number of null overlaps ≥ observed overlap)/(1 + number of permutations). This analysis tested whether the database-annotated target set performed better than genes with similar measurement and study propensity (Supplementary Table S2; Supplementary Data Workbook, sheets “TargetEnrichment,” “MatchedPermutation,” and “PermutationNull”). Target-set overlap and enrichment results are summarized in Table 2.

**Table 2:** Specificity of the database-annotated Lycii Fructus target family for DKD-associated transcriptomic signals.

Outcome	Observed Overlap	Hypergeometric Expectation	Enrichment Ratio	Hypergeometric P	Matched-Null Mean	Empirical P
Glomerular primary DE	16	14.24	1.12	0.347	16.93	0.646
Tubulointerstitial primary DE	24	18.59	1.29	0.102	20.40	0.206
Either compartment primary DE	36	30.06	1.20	0.113	33.14	0.298
Both compartments, same direction	4	1.93	2.07	0.128	2.90	0.325

**Figure 2:**
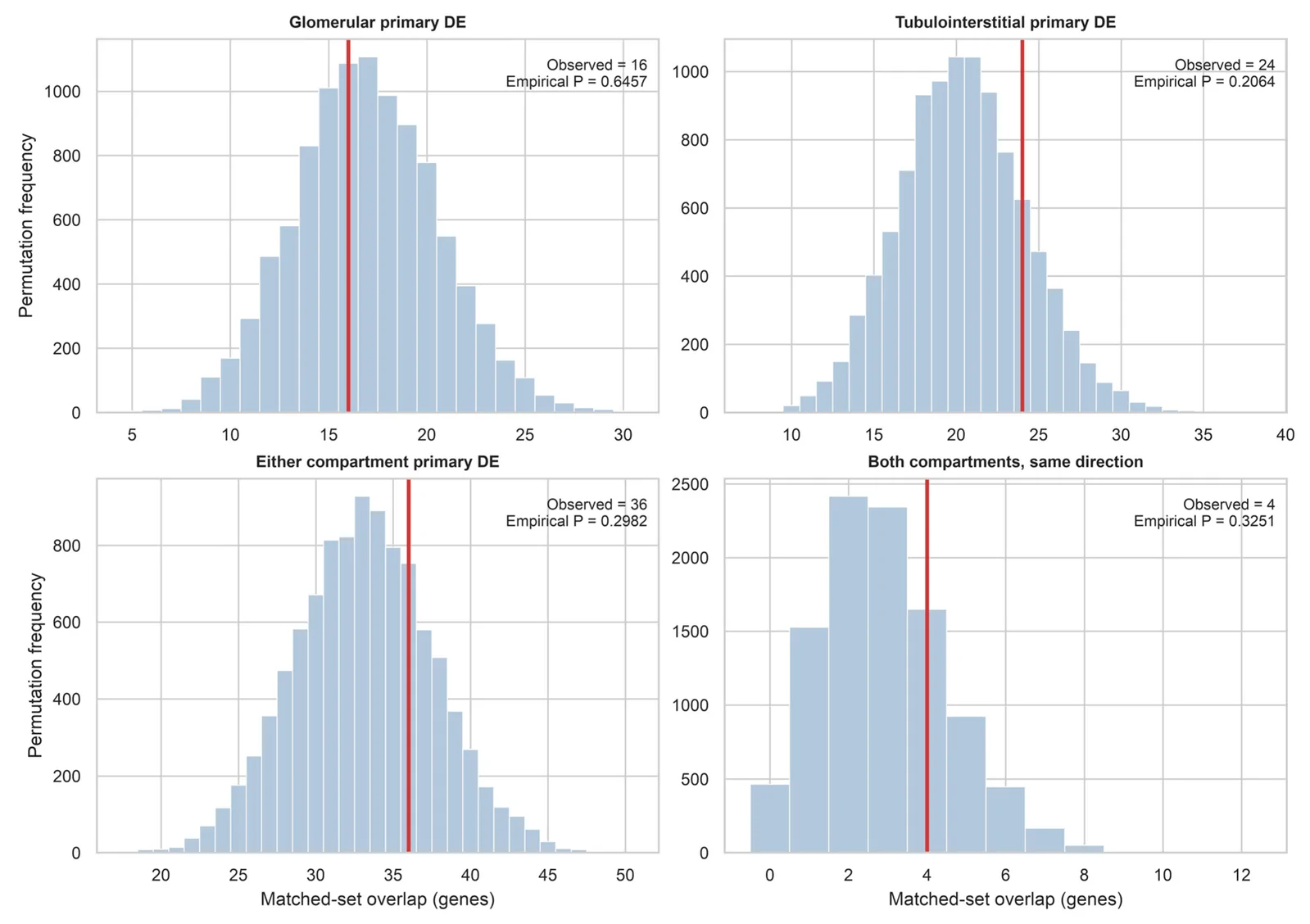
Target-set specificity under matched permutations. Observed overlap counts for the measured database-annotated Lycii Fructus target family are shown against null distributions generated from 10,000 gene sets matched for average expression and the number of unique PubMed identifiers linked through MyGene GeneRIF records. The GeneRIF-derived quantity is a publication-attention proxy, not a total publication count. The vertical line marks the observed count; empirical upper-tail *p* values quantify target-family specificity.

The measured universe comprised 12,501 genes, including 91 of the 106 standardized HERB targets. Matched permutations (10,000 iterations; seed 20260729) controlled for average expression and the number of unique PubMed identifiers linked through MyGene GeneRIF records. The GeneRIF-derived quantity is a publication-attention proxy, not a total publication count. All *p* values are two-sided unless an upper-tail test is explicitly specified. The target-set overlap and enrichment analyses are shown in Figure 2.

### 2.5. External Assessment and Symmetric Evidence Grading

All discovery-positive candidates were assessed in the external cohorts using the same preprocessing and limma framework. Evidence grades were defined before candidate interpretation and applied symmetrically. Tier A required direction concordance with discovery and FDR < 0.05 in both principal external cohorts (GSE96804 and GSE104954-GPL22945). Tier B required direction concordance, FDR < 0.05 in at least one principal external cohort, and nominal *p* < 0.05 in the other. GPL24120 was used only to evaluate directional sensitivity and did not determine the principal tier. Candidates not meeting these criteria were retained in the full evidence table without being promoted as core candidates (Supplementary Tables S3 and S4; Supplementary Data Workbook, sheets “AllCandidateEffects,” “CoreEffects,” and “CoreGrades”).

### 2.6. Per-Cohort Effect Sizes and Expression-Quality Auditing

For the core panel, Hedges’ g and 95% confidence intervals were calculated within each cohort from standardized gene-level expression. Effects were not pooled because the datasets differed in renal compartment, control source, platform, and sampling frame. This decision avoids presenting a single summary estimate as though the cohorts were biologically exchangeable.

*SLC6A2* received additional quality auditing because catecholamine-transporter genes may have low or heterogeneous renal expression. Gene-level mean-expression and variance percentiles were calculated within each dataset. Discovery probe-level effects, direction agreement, and probe-specific FDR values were examined. Present/absent detection calls were not available in the processed series matrices and were therefore not imputed (Supplementary Figure S1; Supplementary Table S6; Supplementary Data Workbook, sheets “SLC6A2GeneQC,” “SLC6A2ProbeSummary,” and “SLC6A2ProbeAudit”). Cross-cohort gene-level statistics are summarized in Table 3.

**Table 3:** Symmetric cross-cohort evidence for the four-gene core panel.

Gene	GSE30528	GSE30529	GSE96804	GPL22945	GPL24120	GPL24120 Direction	Tier
*CASP8*	0.346 (0.031)	0.366 (0.019)	0.281 (0.049)	0.246 (0.012)	0.279 (0.225)	Yes	A
*VEGFA*	−1.443 (5.01 × 10^−^^5^)	−0.482 (0.007)	−0.859 (0.001)	−0.840 (8.56 × 10^−7^)	−0.534 (0.059)	Yes	A
*ADRB2*	0.787 (0.014)	0.892 (0.012)	0.194 (0.054)	0.333 (0.003)	0.063 (0.823)	Yes	B
*SLC6A2*	−0.204 (0.044)	−0.240 (0.021)	−0.559 (2.87 × 10^−5^)	−0.142 (0.109)	−0.101 (0.493)	Yes	B

**Figure 3:**
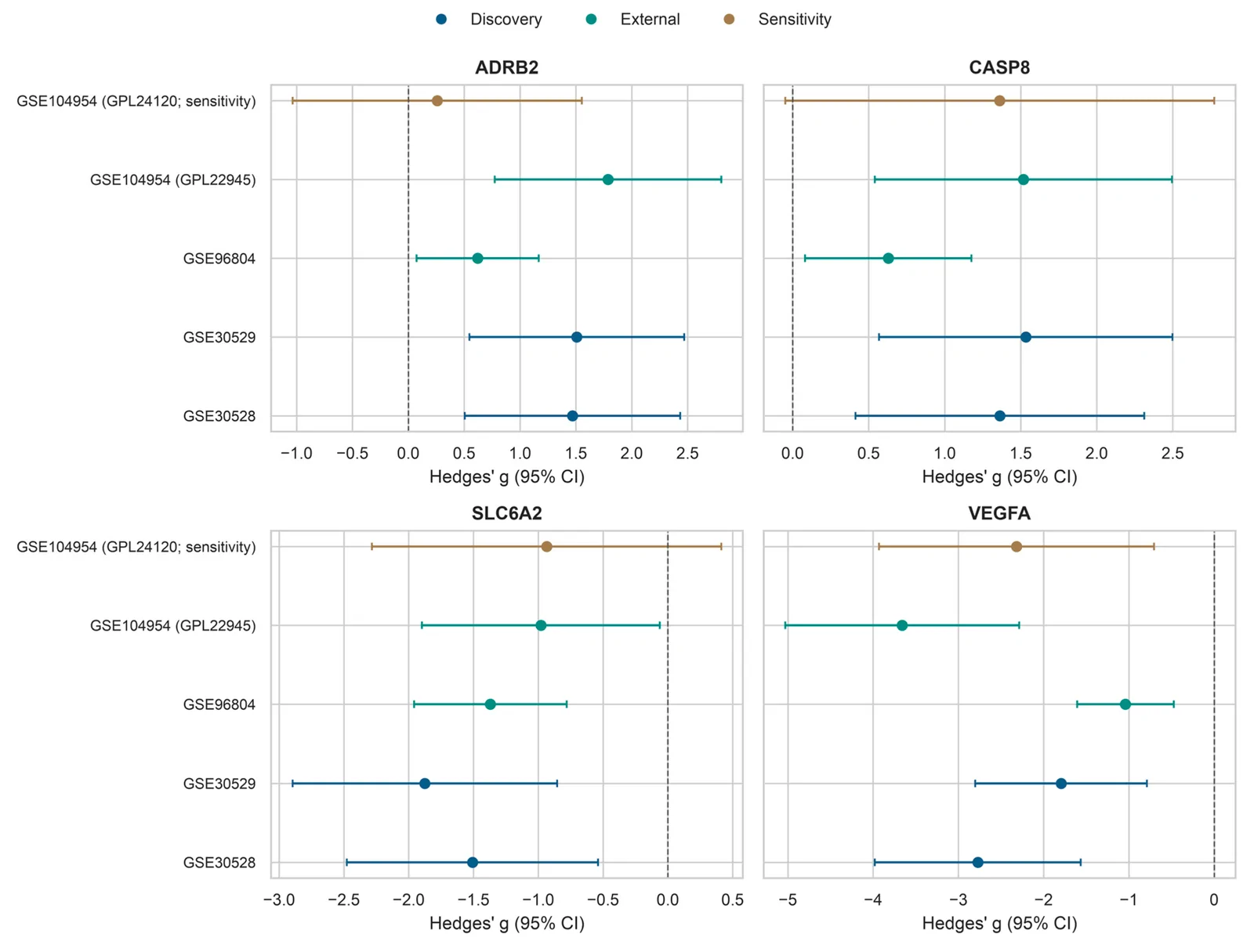
Per-cohort standardized effects for the four-gene core panel. Hedges’ g values are displayed separately for each cohort and renal compartment. Estimates were not pooled across compartments because the cohorts differed in tissue source, platform, control definition, and participant linkage.

**Figure 4:**
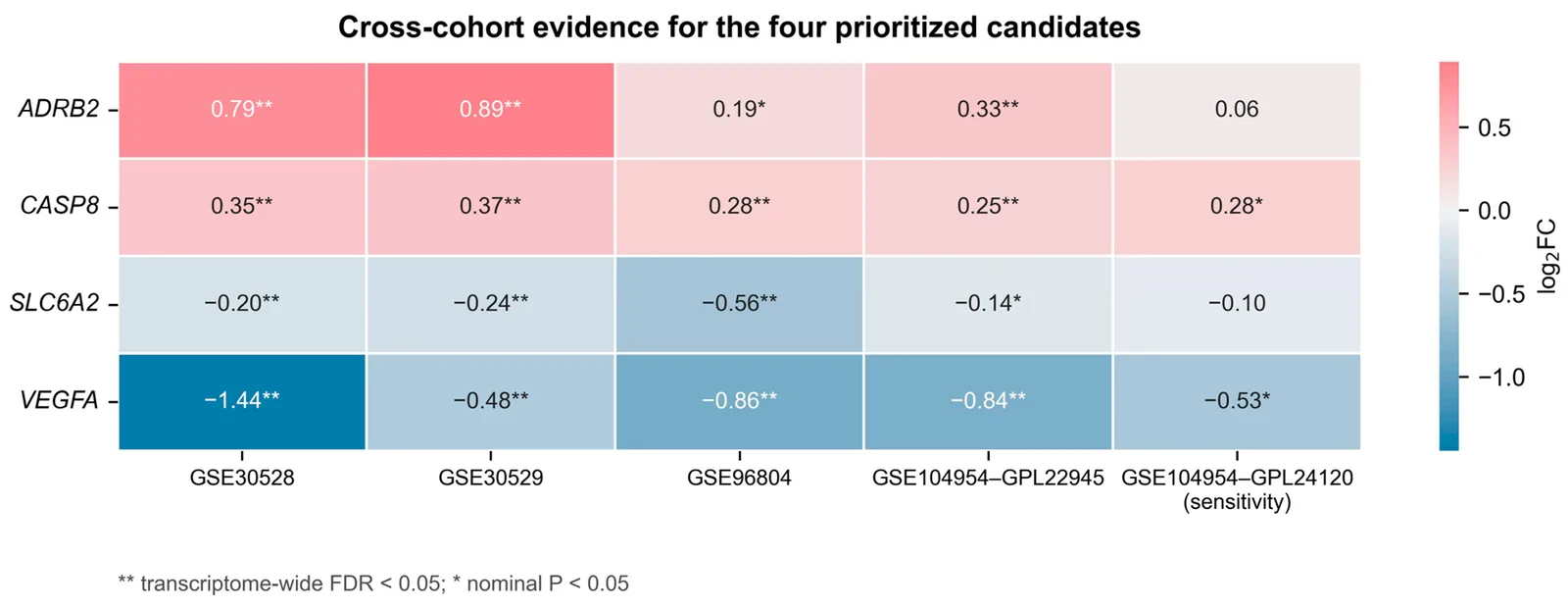
Symmetric cross-cohort evidence matrix for CASP8, VEGFA, ADRB2, and SLC6A2. Color represents log_2_FC, while symbols indicate transcriptome-wide Benjamini–Hochberg FDR support. The same evidence rules were applied to every candidate.

Values are log_2_FC (transcriptome-wide Benjamini–Hochberg FDR). Tier A required direction concordance and FDR < 0.05 in both principal external cohorts (GSE96804 and GSE104954-GPL22945). Tier B required direction concordance in both principal external cohorts and FDR < 0.05 in one, with nominal *p* < 0.05 in the other. GPL24120 was retained as a sensitivity cohort because of its small control group. The corresponding standardized effects and evidence matrix are shown in Figure 3 and Figure 4.

### 2.7. String Network and Functional Context

**Figure 5:**
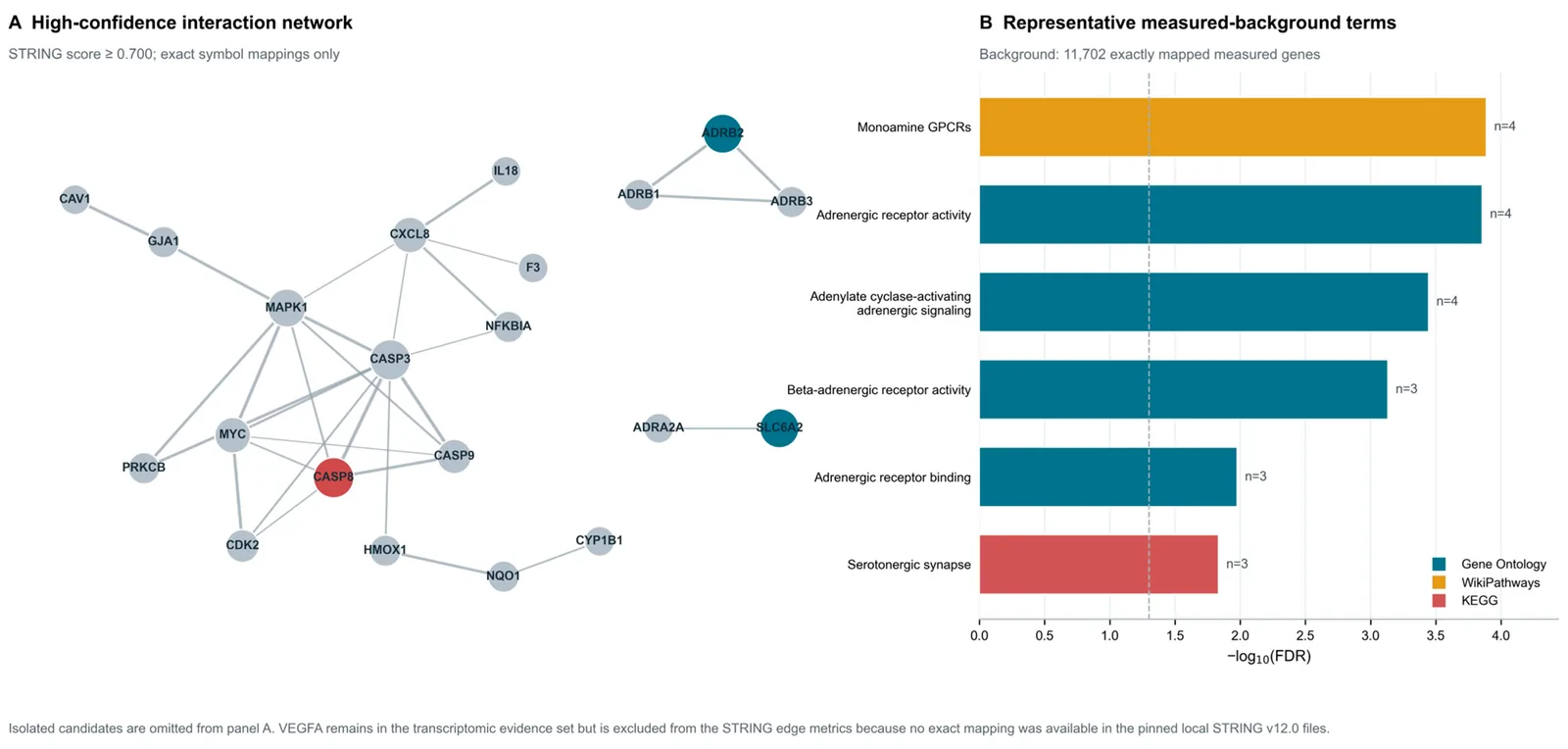
High-confidence STRING network using the measured transcriptomic universe as background. The network provides interaction context for the 36 discovery candidates. VEGFA was excluded from network statistics because the STRING application programming interface resolved the submitted identifier to COL18A1 rather than an exact VEGFA mapping; this event was retained in the mapping audit.

Protein–protein interaction analysis used STRING version 12.0 with a minimum interaction score of 0.700 [26]. The measured transcriptomic universe, rather than the whole genome, was supplied as the enrichment background. Identifier mapping was audited against STRING human alias and protein-information files. Only exact symbol-to-preferred-name mappings were accepted for edge and node metrics. VEGFA was retained in the transcriptomic evidence panel but excluded from STRING edge calculations because the version 12 application-programming interface resolved the input to COL18A1 rather than an exact VEGFA preferred name. Historical mappings were documented but not mixed across releases. Functional interpretation emphasized a DKD-relevant panel of vascular, metabolic, adrenergic, apoptotic, inflammatory, and kidney-injury terms defined before enrichment review (Supplementary Tables S7 and S8; Supplementary Data Workbook, sheets “STRINGMapping,” “VEGFAAudit,” “STRINGEdges,” “STRINGNodes,” “STRINGRelevantTerms,” and “STRINGAllTerms”). The high-confidence STRING interaction network and representative measured-background terms are shown in Figure 5.

### 2.8. Exploratory Candidate-Ranking Sensitivity Analysis

Machine learning was retained only to test the sensitivity of candidate ranking to resampling and preprocessing. Feature selection was repeated within each leave-one-out training fold, followed by L2-regularized logistic regression using the five highest absolute standardized effects in that fold [27]. Models derived separately in the two discovery compartments were transferred to the principal external cohorts after either within-cohort z-score standardization or sample-wise rank normalization. Because the candidate family originated from the same discovery samples, even fold-wise feature selection could not provide an independent estimate of diagnostic performance or generalizability. The analysis was not used for diagnosis, clinical prediction, candidate promotion, or evidence grading (Supplementary Figure S2; Supplementary Table S9; Supplementary Data Workbook, sheets “MLInternal,” “MLExternalZ,” and “MLExternalRank”).

### 2.9. Calibrated Molecular Docking

An exploratory, rule-based screen was used to define the initial ligand set; it was not a drug-likeness or absorption, distribution, metabolism, excretion, and toxicity model. Candidate records received points for the primary HERB-source role (+2), a usable small-molecule structure (+3), a single resolved PubChem compound identifier (CID; +2; multiple CIDs requiring review, +1), defined name or alias matches (+4 to +9), and chemical-class membership (+2 for flavonoids, coumarins, phenolic acids/esters, or sterols; +1 for carotenoids). Molecular weight > 700 Da and absence of a resolved CID incurred penalties of −2 and −4, respectively. A structure was flagged for review when molecular weight was <50 or >750 Da, hydrogen-bond donors exceeded 12, hydrogen-bond acceptors exceeded 18, topological polar surface area exceeded 220 Å^2^, rotatable bonds exceeded 25, heavy atoms exceeded 80, absolute formal charge exceeded 3, or XlogP exceeded 10. Eligibility required a resolved CID, a usable structure without a review flag, a score ≥10, molecular weight ≤ 900 Da, and ≤30 rotatable bonds. Records were deduplicated by PubChem CID, ranked by score, and the first 12 were selected: isoscopoletin, ferulic acid, scopoletin, beta-sitosterol, daucosterol, quercetin, caffeic acid, chlorogenic acid, dihydroisoferulic acid, esculetin, gamma-sitosterol, and Sitosterol alpha1. This transparent prioritization was used only to create a tractable and traceable starting matrix; the score was not interpreted as a pharmacokinetic, pharmacodynamic, or efficacy claim.

Docking was restricted to ADRB2 and SLC6A2 because both had interpretable ligand-binding pockets and available human structures. Candidate ligand structures were obtained from PubChem, target sequences were checked in UniProt, and receptor structures were obtained from the Protein Data Bank [28,29,30]. ADRB2 used PDB 2RH1, and SLC6A2 used PDB 8ZP2 [31,32]. Receptors and ligands were prepared using a consistent protonation and charge workflow. AutoDock Vina was used for docking [33,34]. Target eligibility, all reference-redocking modes, the complete calibrated docking matrix, and the representative pairs shown in the main manuscript are provided in Supplementary Tables S10–S12 and the Supplementary Data Workbook (sheets “DockingTargetAudit,” “RedockingModes,” “CalibratedDocking,” and “SelectedDocking”).

Before herb-derived ligands were interpreted, the crystallographic or deposited reference ligand was removed and redocked into the defined binding site. Calibration was considered acceptable when the top-ranked redocked pose reproduced the reference geometry with root-mean-square deviation (RMSD) < 2.0 Å [35]. Binding affinity and ligand efficiency were reported together. Docking scores were used only to prioritize pose-level structural hypotheses; they were not interpreted as binding constants, efficacy evidence, or proof of a molecular mechanism. The selected calibrated docking results are summarized in Table 4.

**Table 4:** Reference-calibrated molecular docking for selected target-ligand pairs.

Target	PDB	Ligand	Class	Vina Affinity	Ligand Efficiency	Reference	Reference Affinity/RMSD
ADRB2	2RH1	scopoletin	coumarin	−7.95	0.568	CAU	−9.94/1.08
ADRB2	2RH1	quercetin	flavonoid/flavonoid glycoside	−9.79	0.445	CAU	−9.94/1.08
SLC6A2	8ZP2	quercetin	flavonoid/flavonoid glycoside	−8.88	0.404	A1LX4	−8.67/1.09
SLC6A2	8ZP2	caffeic acid	phenolic acid/ester	−7.54	0.580	A1LX4	−8.67/1.09

**Figure 6:**
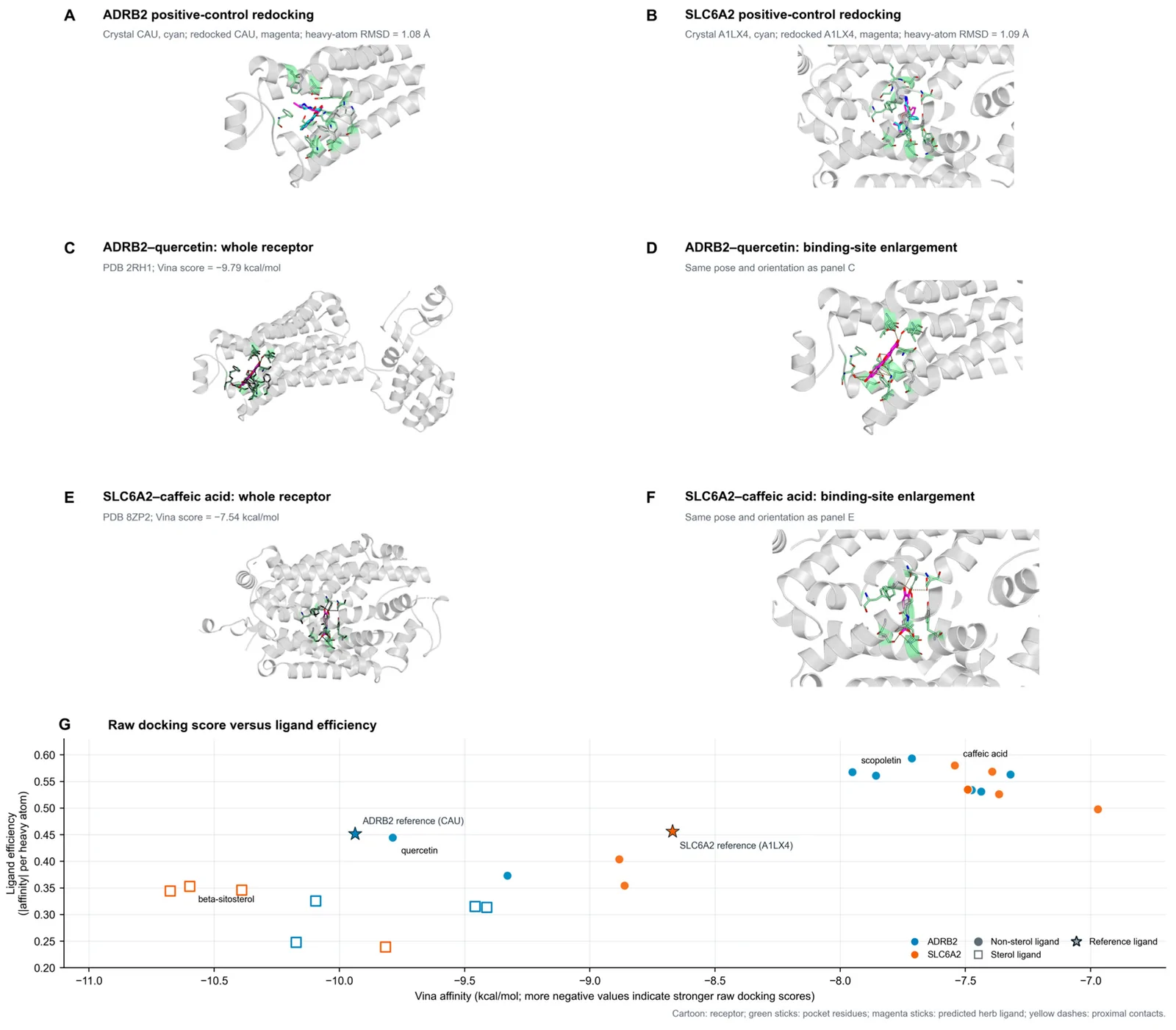
Reference-redocking-calibrated molecular docking. Each target–ligand example contains the whole-protein pose and a magnified view of the same docking pose. In (**panel G**), filled circles denote non-sterol Lycii Fructus-associated ligands, open squares denote sterol ligands, and stars denote the crystallographic reference ligands used for calibration. Reference redocking reproduced carazolol in ADRB2 and atomoxetine in SLC6A2 with RMSD values of 1.08 and 1.09 Å, respectively. Docking is interpreted as structural plausibility rather than evidence of biochemical binding or therapeutic efficacy.

Affinity is reported in kcal/mol; ligand efficiency is |affinity| per heavy atom; RMSD is reported in Å. Reference redocking reproduced carazolol in ADRB2 (PDB 2RH1) and atomoxetine in SLC6A2 (PDB 8ZP2). Docking supports pose-level structural plausibility and does not establish target engagement. The reference-redocking-calibrated docking results are shown in Figure 6.

### 2.10. Statistical Analysis and Reproducibility

All tests were two-sided unless a directional enrichment alternative was explicitly stated. Multiple testing was controlled by the Benjamini–Hochberg procedure. Core transcriptomic and null-model analyses used Python 3.12.13 (NumPy 2.5.1, pandas 3.0.5, and SciPy 1.18.0) and R 4.5.3 with limma 3.66.0. Machine-learning and visualization workflows additionally used scikit-learn 1.8.0, Matplotlib 3.11.1, and seaborn 0.13.2. Docking preparation and scoring used Python 3.11.15, AutoDock Vina 1.2.5, Open Babel 3.1.0, RDKit 2025.09.5, and Meeko 0.7.1. Analyses were conducted with versioned scripts. Exact cohort labels, participant-overlap records, gene-mapping decisions, null-model summaries, candidate-level statistics, software manifests, and figure-source tables are retained with the analysis outputs. Numerical values displayed in the manuscript were populated from these source tables rather than manually re-entered.

## 3. Results

### 3.1. Cohort Structure and Discovery Dependence

The five analyzed dataset/platform subsets contained 143 expression profiles: 44 discovery profiles and 99 external profiles. The 44 discovery arrays represented 35 unique discovery participants because four controls and five participants with DKD appeared in both GSE30528 and GSE30529. Accordingly, 143 denotes the number of analyzed expression profiles and must not be interpreted as 143 independent participants. The two discovery compartments supplied complementary tissue views from a partially overlapping participant set, whereas GSE96804 and GSE104954 were collected separately and provided external assessment under different control sources and platforms.

### 3.2. Discovery Candidates and Target-Set Specificity

Among 12,501 genes in the measured discovery universe, 1956 met the primary criterion in glomeruli and 2554 in tubulointerstitium. Ninety-one HERB-annotated Lycii Fructus targets were measurable. Sixteen targets were discovery positive in glomeruli, 24 in tubulointerstitium, and 36 in at least one compartment; four targets (*ADRB2*, *CASP8*, *SLC6A2*, and *VEGFA*) were significant in both compartments with the same direction.

The overlap did not exceed chance expectations under the specificity tests. Hypergeometric *p* values were 0.347 for glomeruli, 0.102 for tubulointerstitium, 0.113 for either compartment, and 0.128 for same-direction significance in both compartments. In the 10,000 matched permutations, the corresponding empirical *p* values were 0.646, 0.206, 0.298, and 0.325. Thus, the observed target overlap was traceable but not demonstrably enriched beyond expression- and publication-matched genes.

### 3.3. External Evidence Identified a Four-Gene Core Panel

CASP8 and VEGFA met Tier A criteria. *CASP8* was upregulated in the discovery datasets and remained directionally concordant in GSE96804 (log_2_FC = 0.281, FDR = 0.049) and GPL22945 (log_2_FC = 0.246, FDR = 0.012). VEGFA was downregulated in both discovery compartments and in GSE96804 (log_2_FC = −0.859, FDR = 0.001) and GPL22945 (log_2_FC = −0.840, FDR < 0.001).

*ADRB2* and SLC6A2 met Tier B criteria. *ADRB2* was upregulated in discovery, supported in GPL22945 (log_2_FC = 0.333, FDR = 0.003), and nominally significant in GSE96804 (log_2_FC = 0.194, *p* = 0.023, FDR = 0.054). *SLC6A2* was downregulated in discovery, supported in GSE96804 (log_2_FC = −0.559, FDR < 0.001), and nominally significant in GPL22945 (log_2_FC = −0.142, *p* = 0.039, FDR = 0.109). GPL24120 preserved the expected direction for all four genes but had weaker adjusted support, consistent with its small control group.

The primary and strict-threshold findings were distinct. All four genes passed the primary criterion in both discovery compartments. Under the stricter criterion, *ADRB2* passed in both compartments; *VEGFA* passed in glomeruli but not tubulointerstitium; and *CASP8* and *SLC6A2* retained FDR < 0.05 in both compartments but did not reach |log_2_FC| ≥ 0.585 in either compartment (Supplementary Figure S3; Supplementary Table S5). The evidence grades therefore reflect cross-cohort reproducibility under the defined primary criterion, with the stricter threshold reported as sensitivity information rather than substituted post hoc.

Per-cohort standardized effects were directionally stable. *VEGFA* showed negative Hedges’ g estimates in all five datasets, ranging from −1.041 to −3.659. SLC6A2 estimates ranged from −0.935 to −1.875. *ADRB2* and *CASP8* were positive across all datasets, although the GPL24120 confidence intervals were wide. Because compartments and control sources differed, these values are displayed separately rather than combined into a pooled effect.

### 3.4. SLC6A2 Signal Was Directionally Consistent but Measurement-Aware

SLC6A2 occupied moderate mean-expression percentiles in the discovery datasets but low variance percentiles. Seven probes mapped to SLC6A2 on each discovery platform representation. Five of seven probes were negative in GSE30528 and all seven were negative in GSE30529; three and two probes, respectively, met probe-level FDR < 0.05. External cohorts also showed negative gene-level effects. These observations support directional consistency while indicating that SLC6A2 should remain a measurement-aware structural candidate rather than be interpreted from a single probe or a single cohort.

### 3.5. Measured-Background String Analysis Provided Context, Not Target Specificity

Of 12,501 measured genes, 11,702 mapped exactly to STRING version 12 identifiers. Thirty-five of the 36 discovery candidates mapped exactly. The high-confidence candidate network contained 30 edges, and 491 enriched terms met FDR < 0.05 when the measured background was used. Eighteen terms fell within the DKD-relevant interpretive panel. The resulting context involved adrenergic signaling, vascular regulation, metabolic response, apoptosis, and cellular injury. These findings describe connectivity among already selected candidates; they do not reverse the non-significant target-set specificity result.

VEGFA was not assigned a STRING degree value. STRING version 12 resolved the input symbol to COL18A1, which failed the exact preferred-name rule. The transcriptomic VEGFA evidence was retained, but no forced or cross-version network mapping was introduced.

### 3.6. Reference-Redocking Calibration Supported Pose-Level Docking Interpretation

Reference redocking reproduced carazolol in ADRB2 with RMSD = 1.08 Å and atomoxetine in SLC6A2 with RMSD = 1.09 Å. For ADRB2, scopoletin and quercetin yielded docking scores of −7.951 and −9.787 kcal/mol, with ligand efficiencies of 0.568 and 0.445, respectively; the carazolol reference score was −9.937 kcal/mol. For SLC6A2, quercetin and caffeic acid yielded scores of −8.882 and −7.543 kcal/mol and ligand efficiencies of 0.404 and 0.580; the atomoxetine reference score was −8.670 kcal/mol. The calibrated poses identify chemically testable receptor-ligand configurations but do not establish binding or biological activity.

### 3.7. Exploratory Candidate Ranking Was Sensitive to Preprocessing

The exploratory ranking analysis was preprocessing-sensitive. Under within-cohort z-score standardization, external area-under-the-curve estimates were 0.84 for GSE96804 (permutation *p* = 0.091) and 0.98 for GPL22945 (*p* = 0.021); after rank normalization, they decreased to 0.70 (*p* = 0.313) and 0.88 (*p* = 0.119), respectively, while GPL24120 remained sensitivity-only. Because the candidates were discovery-derived and most permutation results were non-significant, this analysis supplied sensitivity context only and did not support a diagnostic or cross-population generalizability claim (Supplementary Figure S2; Supplementary Table S9; Supplementary Data Workbook, sheets “MLInternal,” “MLExternalZ,” and “MLExternalRank”).

## 4. Discussion

### 4.1. Principal Interpretation

This study separated two questions that are often conflated: whether a database-annotated herbal target is altered in DKD, and whether the annotated target set is unusually concentrated among DKD-associated genes. The first question yielded a reproducible four-gene panel. The second yielded no significant enrichment under either analytical or matched null models. The defensible interpretation is therefore that ADRB2, VEGFA, CASP8, and SLC6A2 are DKD-perturbed genes that also carry Lycii Fructus annotations. The data do not support describing the full target set as a Lycii Fructus-specific DKD mechanism.

### 4.2. Biological Context of the Core Candidates

*VEGFA* showed the largest and most consistent negative effects. VEGF signaling participates in glomerular endothelial homeostasis, permeability, and podocyte–endothelial communication, but its role is context-dependent: both excess signaling and excessive inhibition can damage the renal filtration barrier [36,37]. Recent work on VEGF-B further connects vascular signaling with renal lipid handling and DKD progression [38,39]. The present result prioritizes *VEGFA* as a compartment-reproducible marker of DKD-associated transcriptional disturbance; it does not imply that simple activation or inhibition would be therapeutically appropriate.

*ADRB2* and *SLC6A2* place adrenergic and norepinephrine-handling processes within the candidate panel. Renal sympathetic activity can influence vascular tone, sodium handling, and metabolic stress, while norepinephrine transport regulates local catecholamine exposure [40]. *ADRB2* met the stricter discovery threshold in both compartments and showed principal external support. *SLC6A2* was directionally stable but more sensitive to platform and variance characteristics. Their joint interpretation is therefore stronger at the pathway-context level than as proof of a direct herb-receptor interaction.

*ADRB2* and *SLC6A2* are pharmacologically tractable proteins with well-characterized binding sites, illustrated here by the reference ligands carazolol and atomoxetine. Those ligands were used only to calibrate the docking workflow; their inclusion does not support repurposing them for DKD. *VEGFA* and *CASP8* are also biologically actionable, but the likely consequences of intervention are cell-type- and disease-stage-dependent and cannot be inferred from transcript direction alone. Translational follow-up therefore requires target-engagement and renal cell-phenotype experiments before any therapeutic interpretation.

*CASP8* links the panel to regulated cell-death and inflammatory signaling. Apoptotic and pyroptotic processes contribute to podocyte and tubular injury in diabetic nephropathy, with effects that depend on cell type and disease stage [41,42,43]. CASP8’s consistent positive direction across compartments and principal external cohorts supports its Tier A classification. Related enrichment terms involving AGE-RAGE signaling and stress-response mediators such as GDF15 provide additional disease context [44,45], although pathway labels should not be read as independent validation of individual genes.

Taken together, the four candidates delineate complementary rather than interchangeable biological axes: *VEGFA* reflects filtration-barrier and vascular signaling, *CASP8* reflects cell-injury and inflammatory signaling, and *ADRB2/SLC6A2* reflect catecholamine receptor and transporter biology. Their discovery effect sizes were also nonuniform: *ADRB2* met the strict threshold in both compartments, *VEGFA* did so only in glomeruli, and *CASP8/SLC6A2* showed smaller but externally concordant shifts. This pattern argues for candidate-specific follow-up rather than a single undifferentiated “herbal mechanism.” Experiments should first establish renal cell-type localization and disease-context expression, then test direct ligand engagement only for the structurally tractable targets, and finally determine whether perturbing each axis changes DKD-relevant cellular phenotypes. These are testable priorities derived from the computational evidence, not evidence that Lycii Fructus exposure modulates all four genes in vivo.

### 4.3. Why the Null Result Matters

The matched-permutation result changes the weight assigned to the database overlap. HERB targets are enriched for genes that are well studied, connected, and readily measured. Matching on average expression and the GeneRIF-linked PubMed identifier proxy directly addresses two sources of preferential recovery. The lack of significant enrichment does not erase the reproducibility of individual candidates, but it prevents the overlap count from being used as evidence of herbal specificity. This distinction makes the analysis more useful for designing follow-up studies: candidate selection can be based on cross-cohort evidence and structural tractability rather than on the size of a network-pharmacology intersection.

### 4.4. Scope of the Docking Evidence

Docking was performed after transcriptomic prioritization and restricted to two structurally tractable targets. Reference-ligand redocking demonstrated that the search spaces and preparation workflow could reproduce known poses within the conventional 2.0 Å criterion. Reporting ligand efficiency alongside docking score reduced, but did not eliminate, size-related scoring bias. Replicated, adequately converged explicit-membrane molecular-dynamics simulations and a validated free-energy workflow could examine pose retention and interaction persistence, but such analyses were not performed in the present study and would still not establish binding affinity or biological activity. The selected poses are therefore suitable for prioritizing biochemical binding assays or cell-based perturbation studies, not for inferring target engagement, pharmacokinetics, or renal efficacy.

### 4.5. Strengths and Limitations

The analysis used compartment-resolved human tissue, explicitly audited participant overlap, applied the same external evidence rules to all candidates, tested target-set specificity against matched null sets, retained per-cohort effects, used the measured transcriptomic universe for STRING enrichment, and calibrated docking with reference ligands. These design choices make each evidential step traceable.

Several constraints define the inferential boundary. The study was designed to prioritize candidates from public human renal transcriptomes rather than to establish biological causality in a new experimental model; no independent cell, animal, or clinical validation was undertaken. The cross-cohort evidence should therefore be viewed as defining experimentally testable candidates. Renal cell-type localization, disease-context expression, direct target-engagement assays for structurally tractable proteins, and perturbation of DKD-relevant phenotypes would be appropriate next steps.

Some measurement and cohort questions could not be addressed uniformly with the information available in the deposited datasets. Present/absent calls for SLC6A2 were not consistently available across the processed public matrices, so a uniform retrospective detection-call filter was not feasible without introducing non-comparable assumptions. We therefore assessed complementary indicators, including non-missingness, groupwise distributions, expression and variance percentiles, probe-level direction concordance, and cross-cohort consistency, and retained SLC6A2 only as a measurement-aware Tier B candidate. Control provenance also differed by compartment. The principal tubulointerstitial external cohort included living-donor controls, whereas the principal glomerular cohort used histologically unaffected tissue from tumor nephrectomies. A search of the available datasets did not identify a comparable living-donor glomerular cohort for the analyzed contrast; the requested living-donor-only glomerular sensitivity analysis was therefore not feasible with the currently available data. This consideration is most relevant to the magnitude and transportability of the glomerular estimates, particularly for VEGFA, rather than to a pooled effect, which was not calculated.

Other limitations include the small and partially paired discovery datasets, the three controls in GPL24120, and the inability of bulk-tissue expression to identify the contributing renal cell types. Publicly recoverable clinical covariates were incomplete and insufficiently harmonized to support a uniform cross-cohort adjusted analysis, leaving residual differences in demographic and disease characteristics as a possible source of between-cohort variation. HERB annotations combine heterogeneous evidence and do not establish exposure to bioavailable Lycii Fructus constituents. STRING mapping excluded VEGFA from network metrics under an exact version-specific rule, and docking remains a static pose-scoring procedure. These considerations may influence effect magnitude or biological interpretation, but they do not negate the directionally concordant signals observed across the available cohorts. The present study therefore supports a transparent candidate panel and a defined validation path rather than causal attribution or therapeutic efficacy.

## 5. Conclusions

Cross-cohort, renal compartment-aware analysis reduced 106 standardized Lycii Fructus annotations to four reproducible DKD-associated candidates. *CASP8* and *VEGFA* received Tier A external support, and *ADRB2* and *SLC6A2* received Tier B support. The target set as a whole was not enriched beyond expression- and publication-matched expectations. Measured-background network analysis and reference-redocking-calibrated docking supplied biological and structural context without changing that specificity result. *ADRB2*, *VEGFA*, *CASP8*, and *SLC6A2* constitute a transparent panel for subsequent pharmacological testing.

## Data Availability

The original data presented in the study are openly available in the NCBI Gene Expression Omnibus at accession numbers GSE30528, GSE30529, GSE96804, and GSE104954. Additional supporting data—including candidate-level results, participant-overlap records, threshold sensitivity, mapping audits, permutation summaries, machine-learning sensitivity analyses, docking audits, and complete calibrated docking results—are provided in Supplementary Figures S1–S3, Supplementary Tables S1–S12, and the accompanying Supplementary Data Workbook. Versioned analysis scripts and figure-source tables are available from the corresponding authors upon reasonable request.

## Supplementary Materials

The following supporting information can be downloaded at: https://www.mdpi.com/article/10.3390/cimb48080829/s1.
