# Supplementary material for: Cross-Cohort, Renal Compartment-Aware Transcriptomic Assessment of Lycii Fructus-Annotated Targets in Diabetic Kidney Disease
Source: Curr Issues Mol Biol. 2026 Aug 14;48(8):829. doi: 10.3390/cimb48080829 (PMC13510843; doi:10.3390/cimb48080829)
Supplement: Supplementary file 1 [file cimb-48-00829-s001.zip › CIMB_Supplementary_Materials_cimb-4463555_2026-08-04.pdf]

Supplementary Materials for: Cross-Cohort, Renal Compartment-Aware Transcriptomic Assessment of Lycii Fructus-Annotated Targets in Diabetic Kidney Disease

Jinhao Zou [1,2], Xiaowei Tian [2], Ye Sun [1,2],* and Siyi Wang [3],*

1. Key Laboratory of Dryness Syndrome in Chinese Medicine, Ministry of Education, Ningxia Medical University, Yinchuan 750004, China
2. College of Traditional Chinese Medicine, Ningxia Medical University, Yinchuan 750004, China
3. College of Pharmacy, Ningxia Medical University, Yinchuan 750004, China

** Correspondence: sunye123@nxmu.edu.cn (Y.S.); WSY06182021@nxmu.edu.cn (S.W.)*

This file contains Supplementary Methods, Figures S1–S3, and Tables S1–S12. Complete row-level results are provided in the accompanying Excel workbook.

Supplementary Methods

S1. Dataset linkage and unit of analysis

GSE30528 and GSE30529 were audited using the individual identifiers available in the GEO sample metadata. Four control participants and five DKD participants contributed samples to both renal compartments. The two discovery datasets were therefore analyzed separately as linked compartmental views rather than pooled as independent studies. Cross-compartment random-effects estimates and I^2 statistics were not used in the revised evidence grading.

S2. Target standardization and differential-expression criteria

A total of 106 HERB-derived human target annotations were standardized to current gene symbols and mapped to the expression platforms without using phenotype labels during probe aggregation. Differential expression was estimated with limma. The primary discovery criterion was FDR < 0.05 and $|\log_2FC| \geq 0.20$; $|\log_2FC| \geq 0.585$ was examined as a stricter fold-change sensitivity threshold. The two thresholds are reported separately and were not treated as interchangeable.

S3. Target-set specificity analyses

Target-set specificity was evaluated against the 12,501 genes measurable in both discovery datasets. Upper-tail hypergeometric tests quantified overlap with compartment-specific differential-expression outcomes. In parallel, 10,000 matched target sets were sampled after matching genes on mean expression and the number of unique PubMed identifiers linked through MyGene GeneRIF records. This GeneRIF-derived variable was treated as a publication-attention proxy, not a total publication count. The empirical upper-tail P value was calculated as (1 + the number of null overlaps at least as large as the observed overlap)/(1 + 10,000). These analyses test whether the HERB-annotated set is more concentrated among DKD-associated genes than comparable measured genes; they do not test therapeutic efficacy.

S4. Symmetric evidence grading and external cohorts

The same transcriptomic evidence rules were applied to ADRB2, CASP8, SLC6A2, and VEGFA. Tier A required discovery-direction concordance plus FDR < 0.05 in both principal external cohorts. Tier B required discovery-direction concordance, FDR < 0.05 in one principal external cohort, and nominal $P < 0.05$ in the other. GSE96804 and the GSE104954 GPL22945 subset were the principal external cohorts. The GPL24120 subset, which has only three controls and was generated on a different platform, was retained only as a directional sensitivity analysis and did not determine the principal tier.

S5. SLC6A2 expression and probe-level quality control

SLC6A2 was examined at both gene and probe levels. Gene-level checks included non-missing fraction, cohort-specific distribution, mean-expression percentile, and variance percentile. In GSE30528, five of seven mapped probes had negative $\log_2FC$ values; in GSE30529, all seven probes were negative. The complete probe-level audit is supplied in the data workbook so that the aggregate signal can be evaluated independently of a single probe.

S6. STRING mapping, network construction, and enrichment background

STRING v12 was queried using exact human gene-symbol resolution. Candidate interactions were retained at a combined confidence score ≥ 0.700. The 12,501-gene measured universe was supplied as the enrichment background. VEGFA was not force-mapped: the interface returned a non-equivalent COL18A1 identifier for the queried symbol in the recorded release, and that resolution was rejected. Network measures therefore apply only to exactly resolved candidates; this mapping decision is documented rather than interpreted as absence of VEGFA biology.

S7. Exploratory machine-learning sensitivity analyses

Exploratory classifiers used nested leave-one-out feature selection within each discovery cohort. External transfer was evaluated after independent within-cohort z-score standardization and in a rank-based sensitivity analysis. The GPL24120 subset was treated as sensitivity-only. Machine-learning results were retained as secondary robustness analyses and were not used to assign the principal evidence tiers.

S8. Reference-calibrated molecular docking

Docking targets were selected only when a structurally interpretable non-covalent pocket and a suitable reference ligand were available. Reference redocking was accepted when the symmetry-aware heavy-atom RMSD was below 2.0 Å. Herb-derived ligand scores were reported together with heavy-atom-normalized ligand efficiency and the corresponding reference-

ligand values. No molecular-dynamics simulation or MM-PBSA/MM-GBSA calculation was performed. Docking poses were used to assess structural plausibility only and were not interpreted as evidence of binding, activity, or treatment efficacy.

Supplementary Figures

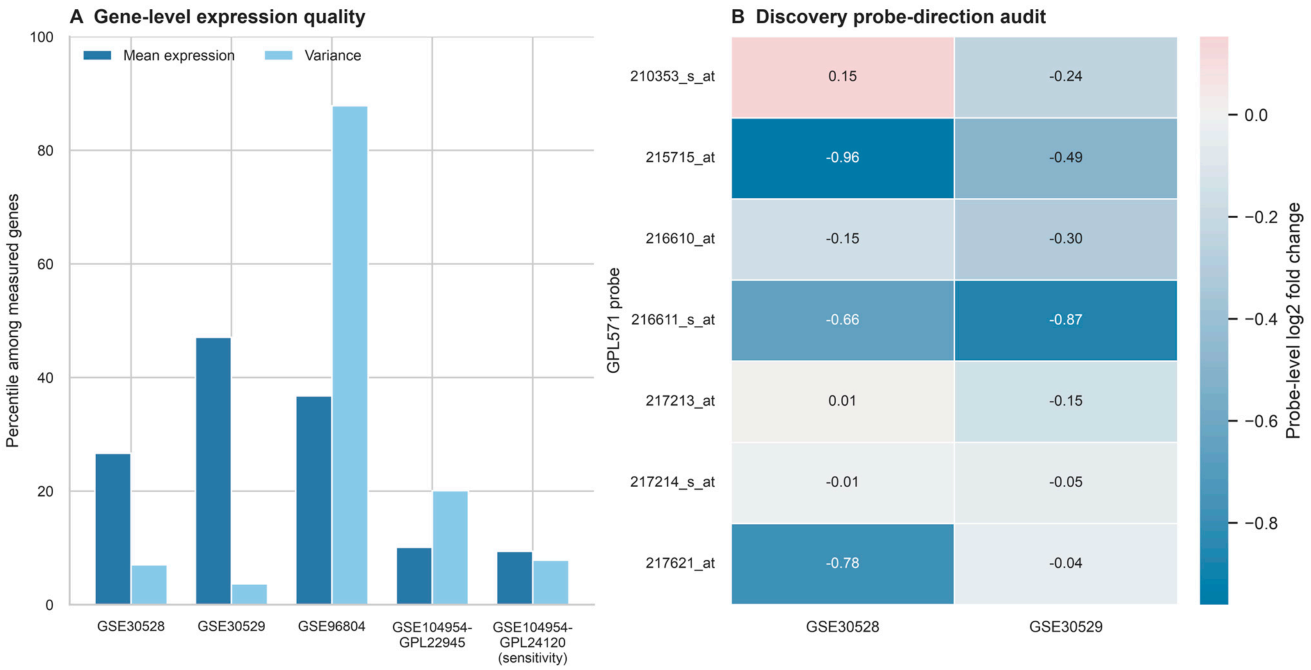

Figure S1. SLC6A2 expression and probe-level quality control. Gene-level distributions are shown by cohort and group, together with discovery probe-level log_2FC estimates. The figure is intended to assess measurability and directional consistency rather than diagnostic performance.

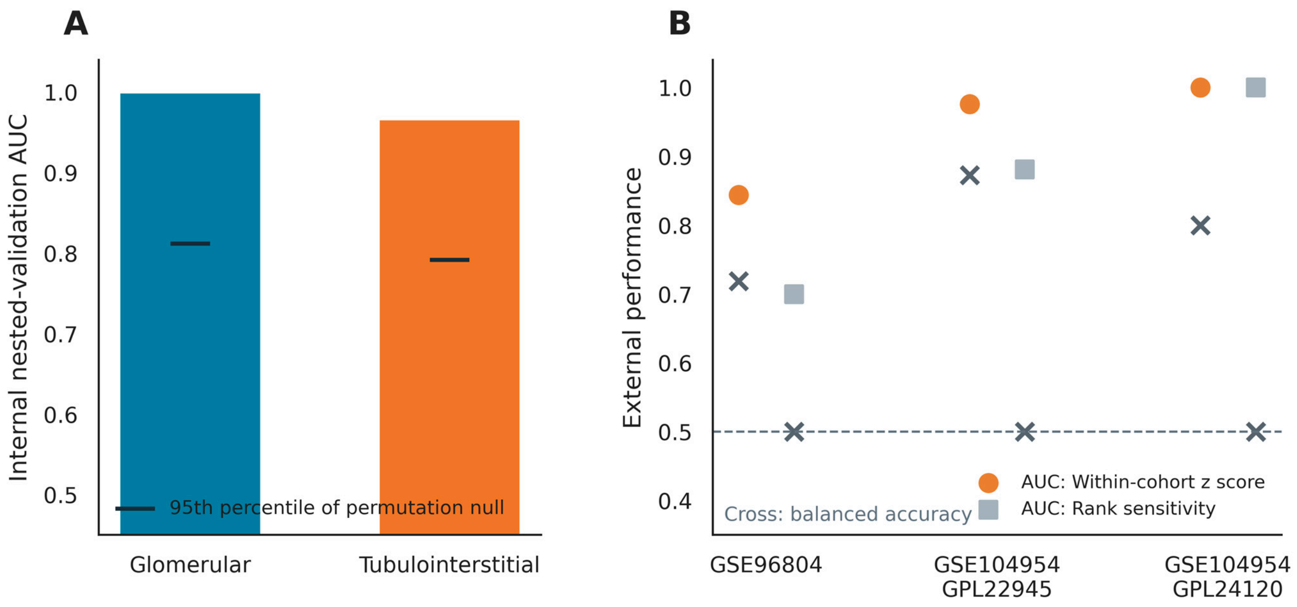

Figure S2. Exploratory nested machine-learning sensitivity analyses. Feature selection was performed within resampling folds. External transfer used the principal external cohorts; GPL24120 is shown only as a sensitivity cohort because it has only three controls and was generated on a different platform.

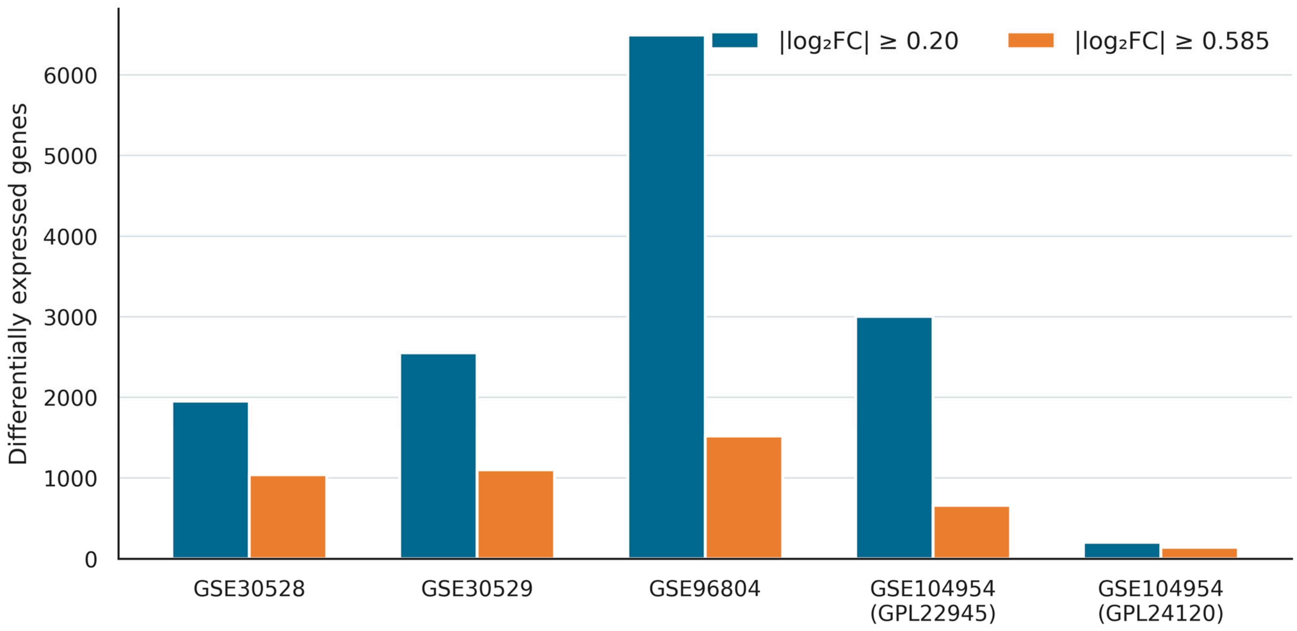

Figure S3. Cohort-level sensitivity of differential-expression counts to the fold-change threshold. Bars show the numbers of genes meeting FDR < 0.05 together with the primary threshold ($|\log_2 FC| \geq 0.20$) or the stricter threshold ($|\log_2 FC| \geq 0.585$). The GPL24120 subset was retained as a sensitivity cohort because it has only three controls and was generated on a different platform.

Supplementary Tables

Table S1. Participant overlap between the two discovery compartments.

Group	GSE30528 unique	GSE30529 unique	Shared n	Shared IDs	Independent ?
Control	13	12	4	76;77;81;82	No
DKD	9	10	5	164;168;178;62;67	No

Note: Shared participants preclude treating the two discovery compartments as independent studies.

Table S2. Target-set specificity against the measured-gene universe.

Outcome	Observed	Expected	Ratio	Hypergeometric P	Matched-null mean	Empirical P
Glomerular primary DE	16	14.24	1.12	0.347	16.93	0.646
Tubulointerstitial primary DE	24	18.59	1.29	0.102	20.40	0.206
Either compartment primary DE	36	30.06	1.20	0.113	33.14	0.298
Both compartments, same direction	4	1.93	2.07	0.128	2.90	0.325

Note: Neither the hypergeometric nor matched-permutation analysis supported target-set enrichment at $P < 0.05$.

Table S3. Per-cohort effect sizes for the four core candidates.

Gene	Dataset	Role	Compartment	DKD n	Control n	Hedges' g	95% CI
ADRB2	GSE30528	Discovery	Glomerular	9	13	1.47	0.51 to 2.43
ADRB2	GSE30529	Discovery	Tubulointerstitial	10	12	1.51	0.55 to 2.47
ADRB2	GSE104954_GPL22945	External	Tubulointerstitial	7	18	1.79	0.77 to 2.80
ADRB2	GSE96804	External	Glomerular	41	20	0.620	0.07 to 1.17
ADRB2	GSE104954_GPL24120	Sensitivity	Tubulointerstitial	10	3	0.259	-1.04 to 1.55
CASP8	GSE30528	Discovery	Glomerular	9	13	1.36	0.41 to 2.31
CASP8	GSE30529	Discovery	Tubulointerstitial	10	12	1.53	0.57 to 2.50
CASP8	GSE104954_GPL22945	External	Tubulointerstitial	7	18	1.52	0.54 to 2.49
CASP8	GSE96804	External	Glomerular	41	20	0.629	0.08 to 1.18
CASP8	GSE104954_GPL24120	Sensitivity	Tubulointerstitial	10	3	1.36	-0.05 to 2.77
SLC6A2	GSE30528	Discovery	Glomerular	9	13	-1.51	-2.48 to -0.54
SLC6A2	GSE30529	Discovery	Tubulointerstitial	10	12	-1.88	-2.90 to -0.85
SLC6A2	GSE104954_GPL22945	External	Tubulointerstitial	7	18	-0.979	-1.90 to -0.06
SLC6A2	GSE96804	External	Glomerular	41	20	-1.37	-1.96 to -0.78
SLC6A2	GSE104954_GPL24120	Sensitivity	Tubulointerstitial	10	3	-0.935	-2.28 to 0.41
VEGFA	GSE30528	Discovery	Glomerular	9	13	-2.77	-3.98 to -1.56
VEGFA	GSE30529	Discovery	Tubulointerstitial	10	12	-1.79	-2.80 to -0.79
VEGFA	GSE104954_GPL22945	External	Tubulointerstitial	7	18	-3.66	-5.03 to -2.29
VEGFA	GSE96804	External	Glomerular	41	20	-1.04	-1.61 to -0.47
VEGFA	GSE104954_GPL24120	Sensitivity	Tubulointerstitial	10	3	-2.32	-3.93 to -0.70

Note: Effect sizes are reported per cohort. No cross-compartment pooled estimate or I^2 statistic was calculated.

Table S4. Symmetric transcriptomic evidence grading.

Gene	Discovery concordant	External concordant	External FDR<0.05 n	External nominal P<0.05 n	Tier	GPL24120 sensitivity
CASP8	Yes	Yes	2	2	A	Yes
VEGFA	Yes	Yes	2	2	A	Yes
ADRB2	Yes	Yes	1	2	B	Yes
SLC6A2	Yes	Yes	1	2	B	Yes

Note: Tier A and Tier B were assigned with identical rules across all four genes. GPL24120 did not determine the principal tier.

Table S5. Strict fold-change threshold sensitivity.

Gene	GSE30528 log_2FC	Primary	Strict	GSE30529 log_2FC	Primary	Strict	Same direction
ADRB2	0.787	Yes	Yes	0.892	Yes	Yes	Yes
CASP8	0.346	Yes	No	0.366	Yes	No	Yes
SLC6A2	-0.204	Yes	No	-0.240	Yes	No	Yes
VEGFA	-1.44	Yes	Yes	-0.482	Yes	No	Yes

Note: The strict criterion was $|log_2FC| \geq 0.585$ with FDR < 0.05.

Table S6. SLC6A2 discovery probe-level directional summary.

Dataset	Compartment	Mapped probes	Negative	Positive	FDR<0.05	Median log_2FC	Negative fraction
GSE30528	Glomerular	7	5	2	3	-0.152	0.714
GSE30529	Tubulointerstitial	7	7	0	2	-0.236	1.00

Note: Complete probe-level estimates and gene-level distribution checks are provided in the Excel workbook.

Table S7. VEGFA STRING v12 identifier-resolution audit.

Query	Release	Source	Observed resolution	Decision	Source SHA-256
VEGFA	12.00	9606.protein.aliases.v12.0.txt.gz and 9606.protein.info.v12.0.txt.gz	Absent from both downloadable human files	Do not force-map; retain transcriptomic evidence but exclude from STRING edge metrics	aliases=b65f730b993ed0c1bd72edf4565d3d425db42861101b29699704810e8f125680; info=144de4b0d98c6a7dfde6ddc2591cf88657f27b989eadff4f501450c3ed1f0f1c
VEGFA	12.00	STRING get_string_ids API	9606.ENSP00000352798 / COL18A1	Rejected because the preferred name did not match the input symbol	Not applicable (API response retained in analysis cache)
VEGFA	11.50	9606.protein.aliases.v11.5.txt.gz	9606.ENSP00000478570	Historical mapping documented only; not mixed into the v12.0 analysis	130e2a1018c559e8c41959b4f8fe703d35bf78aeea784be9d19da7a9f287fd47
ENSP00000330387	12.00	9606.protein.aliases.v12.0.txt.gz and 9606.protein.info.v12.0.txt.gz	Absent from both downloadable human files	Reviewer-suggested identifier not used because it was not supported by the pinned release	aliases=b65f730b993ed0c1bd72edf4565d3d425db42861101b29699704810e8f125680; info=144de4b0d98c6a7dfde6ddc2591cf88657f27b989eadff4f501450c3ed1f0f1c

Note: The non-equivalent COL18A1 resolution was rejected; VEGFA was not assigned zero degree.

Table S8. Selected STRING functional terms using the measured-gene background.

Category	Term	Genes	Background	FDR	Context
WikiPathways	Monoamine GPCRs	4	32	1.30e-04	adrenergic_neurotransmitter
Function	Adrenergic receptor activity	4	9	1.40e-04	adrenergic_neurotransmitter
Process	Adenylate cyclase-activating adrenergic receptor signaling pathway	4	20	3.60e-04	adrenergic_neurotransmitter
Function	Beta-adrenergic receptor activity	3	3	7.40e-04	adrenergic_neurotransmitter
Function	Adrenergic receptor binding	3	18	0.011	adrenergic_neurotransmitter
KEGG	Serotonergic synapse	3	92	0.015	adrenergic_neurotransmitter
PMID	(2022) Network pharmacology and molecular docking reveal zedoary turmeric-trisomes in Inflammatory bowel disease with intestinal fibrosis.	9	36	4.24e-09	cell_injury_stress_inflammation
PMID	(2023) Effects and Mechanisms of Kaempferol in the Management of Cancers through Modulation of Inflammation and Signal Transduction Pathways.	10	106	3.03e-07	cell_injury_stress_inflammation

Category	Term	Genes	Background	FDR	Context
PMID	(2022) Morin encapsulated chitosan nanoparticles (MCNPs) ameliorate arsenic induced liver damage through improvement of the antioxidant system and prevention of apoptosis and inflammation in mice.	7	23	4.13e-07	cell_injury_stress_inflammation
PMID	(2009) Molecular targets of nutraceuticals derived from dietary spices: potential role in suppression of inflammation and tumorigenesis.	9	82	6.36e-07	cell_injury_stress_inflammation
PMID	(2022) Natural Compounds Affecting Inflammatory Pathways of Osteoarthritis.	8	51	7.14e-07	cell_injury_stress_inflammation
PMID	(2018) Early Life Stress and Epigenetics in Late-onset Alzheimers Dementia: A Systematic Review.	13	333	9.11e-07	cell_injury_stress_inflammation
PMID	(2022) The blood–brain barrier and the neurovascular unit in subarachnoid hemorrhage: molecular events and potential treatments.	13	292	3.81e-07	renal_diabetes_vascular_metabolic
PMID	(2023) Novel wine in an old bottle: Preventive and therapeutic potentials of andrographolide in atherosclerotic cardiovascular diseases.	10	122	5.61e-07	renal_diabetes_vascular_metabolic
PMID	(2022) Mechanism of tonifying-kidney Chinese herbal medicine in the treatment of chronic heart failure.	9	85	7.14e-07	renal_diabetes_vascular_metabolic
PMID	(2022) Beneficial Health Effects of Glucosinolates-Derived Isothiocyanates on Cardiovascular and Neurodegenerative Diseases.	10	144	1.29e-06	renal_diabetes_vascular_metabolic
PMID	(2021) The Potential Role of Curcumin in Modulating the Master Antioxidant Pathway in Diabetic Hypoxia-Induced Complications.	8	67	2.53e-06	renal_diabetes_vascular_metabolic
PMID	(2022) Tetramethylpyrazine: An Active Ingredient of Chinese Herbal Medicine With Therapeutic Potential in Acute Kidney Injury and Renal Fibrosis.	7	37	2.53e-06	renal_diabetes_vascular_metabolic

Note: The complete 491-term output and candidate-network node metrics are supplied in the Excel workbook.

Table S9. Exploratory machine-learning summary.

Cohort	Analysis	n	ROC AUC	Permutation P	Status
GSE30528_glomerular	Internal nested LOOCV	22	1.00	1.00e-03	Principal discovery cohort
GSE30529_tubulointerstitial	Internal nested LOOCV	22	0.967	5.00e-04	Principal discovery cohort
GSE96804_glomerular	External z-score transfer	61	0.844	0.091	Principal external cohort
GSE104954_GPL22945_tubulointerstitial	External z-score transfer	25	0.976	0.021	Principal external cohort
GSE104954_GPL24120_tubulointerstitial	External z-score transfer	13	1.00	0.032	Sensitivity only

Note: These analyses were secondary and did not determine the transcriptomic evidence tiers. Rank-based sensitivity results are in the Excel workbook.

Table S10. Docking target eligibility audit.

Gene	Evidence class	Primary status	Reason
ADRB2	cross-compartment externally supported	included	Non-covalent co-crystal reference ligand CAU permits redocking validation.
SLC6A2	cross-compartment directionally supported	included	Non-covalent structure-bound ligand A1LX4 permits redocking validation.
CASP3	tubulointerstitial externally supported	excluded	PDB 2XYG records a covalent LINK between CYS163 SG and TQ8 C22; ordinary non-covalent Vina redocking is not an appropriate validation.
CASP8	cross-compartment externally supported	excluded	PDB 3KJN records a covalent LINK between CYS360 SG and B93 C1; ordinary non-covalent Vina redocking is not an appropriate validation.
BCL2	not retained	excluded	BCL2 was not retained among the 36 revised discovery candidates after phenotype-independent probe-to-gene collapse and gene-level limma analysis.
VEGFA	cross-compartment externally supported	excluded	VEGFA is a secreted growth factor dominated by protein–protein interaction biology and lacks a comparable validated small-molecule pocket in this workflow.

Note: Docking eligibility reflects structural interpretability and reference-ligand availability, not target importance alone.

Table S11. Top-ranked reference-ligand redocking poses.

Gene	PDB	Reference ligand	Mode	Affinity (kcal/mol)	RMSD (Å)	RMSD<2 Å
ADRB2	2RH1	CAU	1	-9.94	1.08	Yes
SLC6A2	8ZP2	A1LX4	1	-8.67	1.09	Yes

Note: Mode 1 is the top-ranked AutoDock Vina pose and is the calibration result reported in the main text. All redocking modes are retained in the Excel workbook.

Table S12. Selected reference-calibrated herb-derived ligand docking results.

Gene	PDB	Ligand	Class	Affinity	Ligand efficiency	Reference	Reference efficiency	Redocking RMSD (Å)
ADRB2	2RH1	scopoletin	coumarin	-7.95	0.568	CAU	0.452	1.08
ADRB2	2RH1	quercetin	flavonoid/flavonoid glycoside	-9.79	0.445	CAU	0.452	1.08
SLC6A2	8ZP2	quercetin	flavonoid/flavonoid glycoside	-8.88	0.404	A1LX4	0.456	1.09
SLC6A2	8ZP2	caffeic acid	phenolic acid/ester	-7.54	0.580	A1LX4	0.456	1.09

Note: Reference RMSD and score values correspond to the top-ranked AutoDock Vina pose (mode 1), consistent with the main text. Docking scores and ligand efficiency are comparative computational descriptors; they do not establish binding or biological activity. All 24 calibrated ligand results are supplied in the Excel workbook.
